# Docking Studies of Phthalimide Pharmacophore as a Sodium Channel Blocker

**Published:** 2013-09

**Authors:** Maryam Iman, Atefeh Saadabadi, Asghar Davood

**Affiliations:** 1 Chemical Injuries Research Center, Baqiyatallah University of Medical Sciences, Tehran, Iran; 2 Department of Medicinal Chemistry, Pharmaceutical Sciences Branch, Islamic Azad University, Tehran, Iran

**Keywords:** Anticonvulsant, Docking, Molecular modeling, Na channel, Phthalimide

## Abstract

***Objective(s):*** Recently, phthalimide derivatives were designed based on ameltolide and thalidomide as they possess a similar degree of anticonvulsant potency due to their phenytoin-like profile. The ability of phthalimide pharmacophore to interact with neuronal voltage-dependent sodium channels was studied in the batrachotoxin affinity assay. Therefore, in the present study, a series of 19 compounds of phthalimide pharmacophore possessing a variety of substituents (NO_2_, NH_2_, Me, Cl, COOH, MeO) at 2-, 3-, and 4- position of the N-phenyl ring and N-(3-amino-2-methylphenyl) succinimide, were subjected to docking studies in order to inhibit voltage-gated sodium channels.

***Materials and Methods*** *:* Chemical structures of all compounds were designed using HYPERCHEM program and Conformational studies were performed through semi-empirical molecular orbital calculations method followed by PM3 force field. Total energy gradient calculated as a root mean square (RMS) value, until the RMS gradient was 0.01 kcal mol^-1^. Among all energy minima conformers, the global minimum of compounds was used in docking calculations. Using a model of the open pore of Na channels, docking study was performed by AUTODOCK4.2 program.

***Results*** *:* Docking studies have revealed that these types of ligands interacted mainly with II-S6 residues of NaV1.2 through making hydrogen bonds and have additional hydrophobic interactions with domain I, II, III and IV in the channel's inner pore.

***Conclusion:*** These computational studies have displayed that these compounds are capable of inhibiting Na channel, efficiently.

## Introduction

Epilepsy is one of the most common neurological disorders, affecting about 1% of the world population. During recent years, many efforts devoted to the development of novel therapeutics have resulted in the availability of several newer drugs as promising anticonvulsants (1, 2). However, the currently available antiepileptic drugs are effective in reducing the severity and number of seizures in less than 80% of patients (3). Moreover, their usage is associated with undesirable side-effects from cosmetic (gingival hyperplasia) to life threatening (e.g. hepatotoxicity, megaloblastic anemia) (4). Therefore, development of new antiepileptic drug with approved therapeutic properties is an important challenge for medicinal chemists.

Sodium channel is one of the best targets in the treatment of epilepsy. Neuronal voltage-gated sodium channels (NVSC) play an important role in the generation and propagation of action potentials in neurons and other excitable cells. Thus, NVSC blocking agents represent a clinically important class of drugs used in the treatment of pain, seizures and arrhythmia. Voltage-gated sodium channels normally consist of an alpha subunit that forms the ion conduction pore and one or two beta subunits that have several functions including modulation of channel gating (5). Expression of the alpha subunit alone is sufficient for production of a functional channel. The alpha-subunit has four repeated domains, labeled I - IV, each containing six membrane-spanning regions, labeled S1 - S6. The family of sodium channels has nine known members. The proteins of these channels are named Na_v_1.1 - Na_v_1.9 (6, 7).

Phthalimide pharmacophore is one of the new ligands that act as sodium channel antagonist, which was designed and evaluated as anticonvulsant agents based on the structure-activity relationships of 4-aminobenzamide derivatives (especially ameltolide) and thalidomide, Vamecq and coworkers studied N-phenyl phthalimide derivatives as rigidified analogues of ameltolide. They designed the 4- amino-N-(2, 6-dimethylphenyl) phthalimide model and subsequent phthalimide pharmacophore without the 4-amino group in the phthaloyl moiety (8, 9). Similarly to ameltolide, N-phenylphthalimide derivatives exhibit a phenytoin-like profile i.e. the interaction with neuronal voltage-dependent sodium channels was studied in the batrachotoxin affinity assay. They are quite potent in the maximal electroshock seizure (MES) test and inactive in the subcutaneous pentylenetetrazole (ScMet) test (9).

 Docking studies are used at different stages in drug discovery such as in prediction of docked structure of ligand-receptor complex and also to rank the ligand molecules based upon their binding energy. Docking protocols aid in elucidation of the most energetically favorable binding pose of a ligand to its receptor. The objective of our current docking study is to elucidate the mode of interaction of phthalimide pharmacophore derivatives with sodium channel (10). In the present study, we report the molecular modeling and drug-receptor interaction profile of 19 phthalimide derivatives which had been designed and synthesized before. It was confirmed that this type of ligand acts as a sodium channel blocker, (8) so we use a model of the open pore of the Na channel as a receptor. This open pore model was developed recently based on homology modeling of the crystal structures of K channels (11).

## Materials and Methods

***Molecular mdeling***

The chemical structures of inhibitors, shown in Table 1 were designed using Hyperchem software (version 7, Hypercube Inc.). Conformational analysis of the desired compounds was performed through Semi-empirical molecular orbital calculations (PM3) method using HYPERCHEM software. The molecular structures were optimized using Polak-Ribiere (conjugate gradient) algorithm until the root mean square (RMS) gradient was 0.01 kcal mol^-1^. Among all energy minima conformers, the global minimum of compounds were used in docking calculations and the resulted geometry was transferred into Autodock (version 4.2) program package, which was developed by Arthur J. Olson Chemometrics Group (12). The structure of docked *N*-phenyl substituent of phthalimide (1-16),* N*-[3-methyl-(2-pyridinyl)] phthalimide (19) and *N*-(3-amino-2-methylphenyl) succinimide (20) are shown in Table 1. 

***Docking***

Docking calculations were performed using Autodock software (version 4.2). A model of Na channel open pore was used as a receptor. This open pore model was developed based on homology model of the crystal structures of K channels (11). The model constructed by homology with potassium channel structures was reasonably successful in accounting for inner pore residue interactions with local anesthetics and anticonvulsant drugs like phenytoin. Desired compounds were docked into the active site as well as phenytoin which were acting as our reference drug and validation of our technique.

Docking was done using AutoDock4.2, in order to assign the perfect grid of each ligand, grid box values were obtained from trial and error and previous studies (13-15). Grid maps with 60×60×60 points were constructed and the grid point spacing was 0.375 Å (16). The implementing Lamarckian Genetic Algorithm (LGA), considered as one of the best docking methods available in AutoDock, was adopted to perform the molecular docking studies. The parameters for LGA were defined as follows: a maximum number of 250,000 energy evaluations; a maximum number of generations of 27,000; and mutation and crossover rates of 0.02 and 0.8, respectively. Pseudo-Solis & Wets parameters were used for local search, and 300 iterations of Solis & Wets local search were imposed. Both Autogrid and Autodock computations were performed on Cygwin and ten independent docking runs were performed for each phthalimide. Final docked conformations were clustered using a tolerance of 1 A ˚ root mean square deviation (RMSD) and the docking log (dlg) files were analyzed using the AutoDock Tools, graphical user interface of Autodock. The docked conformations of each ligand were ranked into clusters based on the binding energy and the top ranked conformations were visually analyzed. Hydrogen bonding and hydrophobic interactions between docked potent agents and macromolecule were analyzed using Auto Dock Tools (version1.50).

## Results

Flexible docking of all data sets used for the computational study, (12) was carried out on the active site of the open pore of the Na channel. Based on the procedure explained in the experimental section, the predicted binding energy, docked energy, and inhibition-constant (Ki) of these inhibitors into the active site are listed in Table 2. The predicted binding and docked energies are the sum of the intermolecular energy and the torsional free-energy penalty, and docking the ligand’s internal energy, respectively, and the inhibition-constant (Ki) is calculated in AutoDock4 as exp (DG × 1000)/(Rcal × TK) where DG is the docking energy, Rcal is 1.98719, and TK is 298.15 (13,16). Our docking results reveal that based on the predicted binding energy, compounds 4, 8, 15, 6, 7, 13 and 14 with -6.46, -6.30, -6.09, -6.02, -5.90, -5.88 and -5.84 kcal/mol binding energy, respectively, are more potent than phenytoin with -5.83 kcal/mol binding energy, as a reference drug. Based on the binding energy, compounds 1, 2, 3, 5, 9, 10, 11, 12, 16, 17, 18, 19 and 20 are less active than phenytoin. According to the K_i_, compounds 4, 8, 15, 6, 7, 13 and 14 with 18.46, 24.13, 34.64, 38.89, 47.64, 48.70 and 52.15 µm inhibition-constant can inhibit the enzyme more efficiently when compared to phenytoin with 53.37µm inhibition-constant.

**Figure 1:**
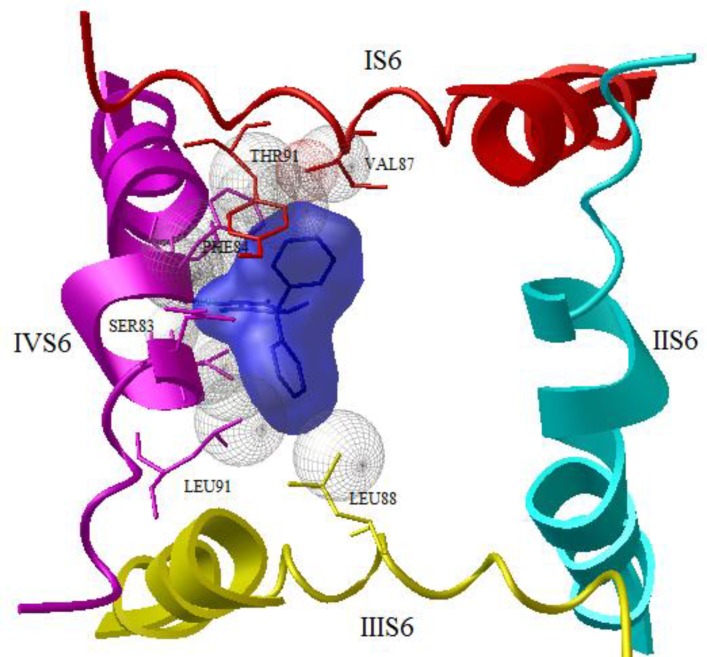
Docked structure of phenytoin in model of the open sodium channel (Nav1.2). The backbones of S6 α-helices of domains I-IV are shown in red, Cyano, yellow and magenta, respectively. Hydrogen bond (distance: 2.06 and binding energy: -5.83) is formed between hydrogen of imide and SER83 of domain IV-S6

**Figure 2:**
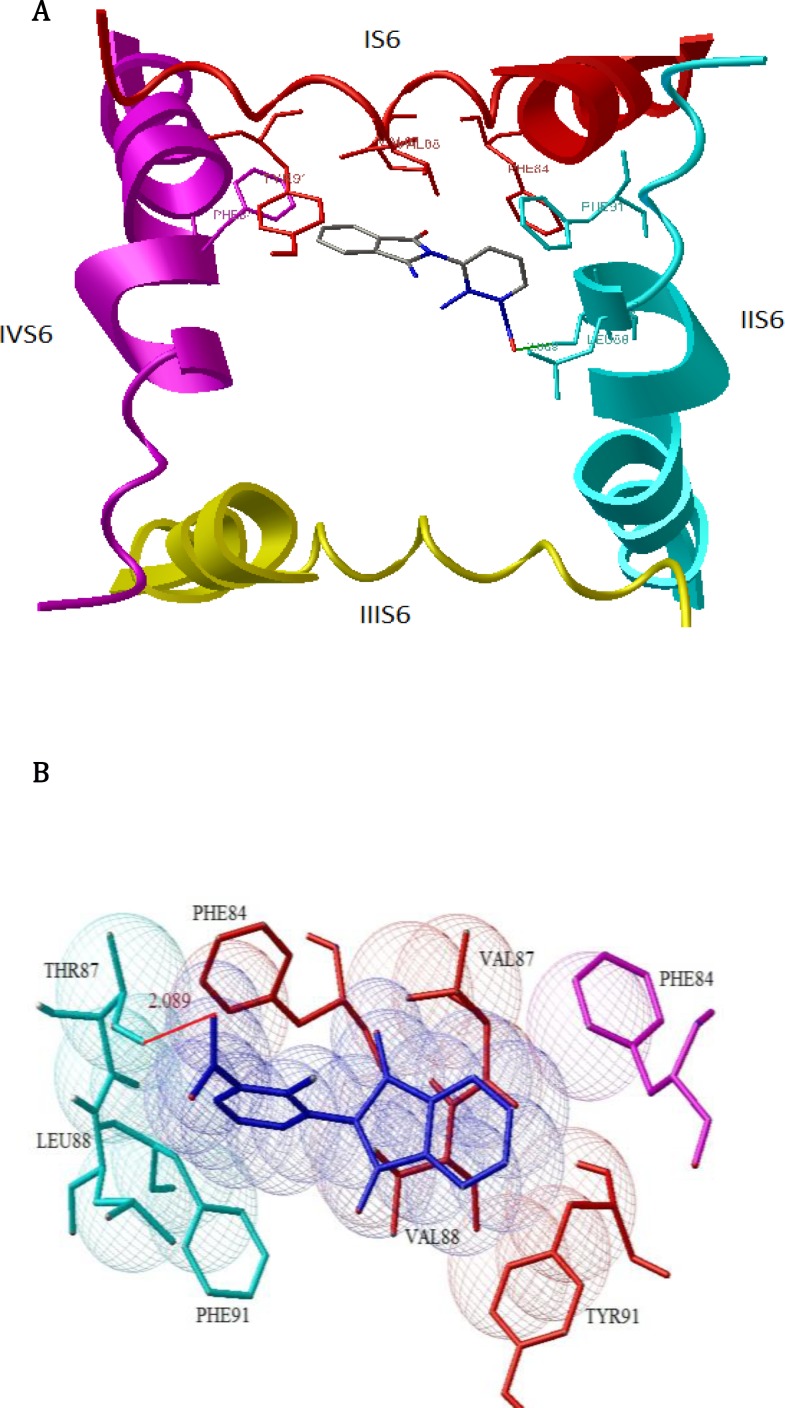
A) Docked structure of compound 4 in model of the open sodium channel (Nav1.2) (top view). The backbones of S6 α-helices of domains I-IV are shown in red, Cyano, yellow and magenta, respectively. B) Docked structure of 4 in model of sodium channel. Hydrogen bonds (distance: 2.088, binding energy: -6.46) are represented in red lines

**Figure 3:**
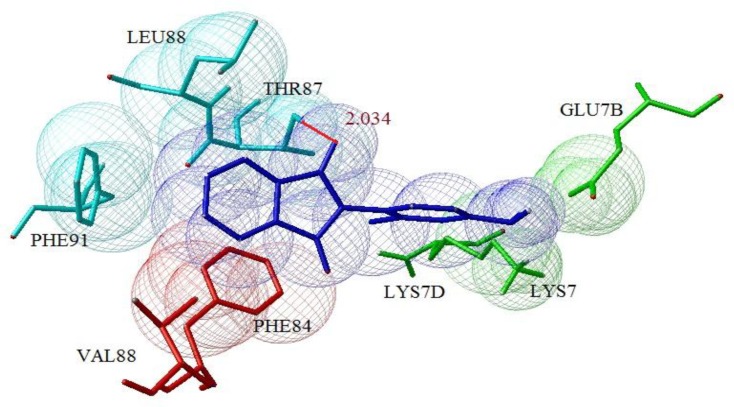
Docked structure of compound 15 in model of sodium channel, hydrogen bond (distance: 2.034, binding energy: -6.09) is represented by dashed red line

**Figure 4:**
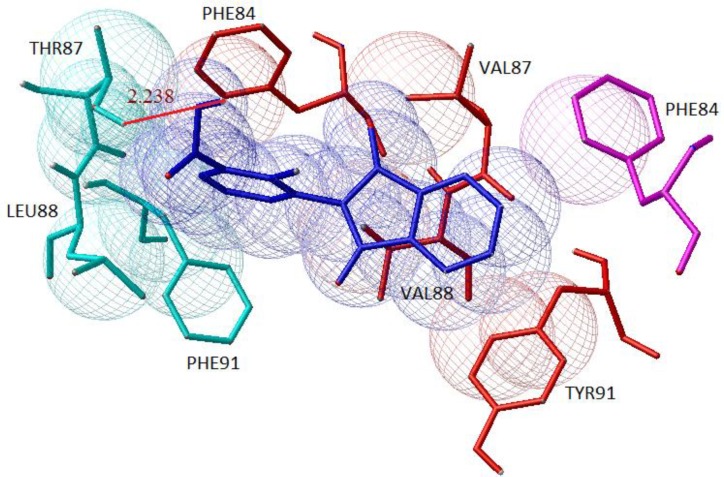
Docked structure of compound 13 in model of sodium channel, hydrogen bond (distance: ‎‎2.238, binding energy: -5.88) is represented by dashed red line

**Figure 5:**
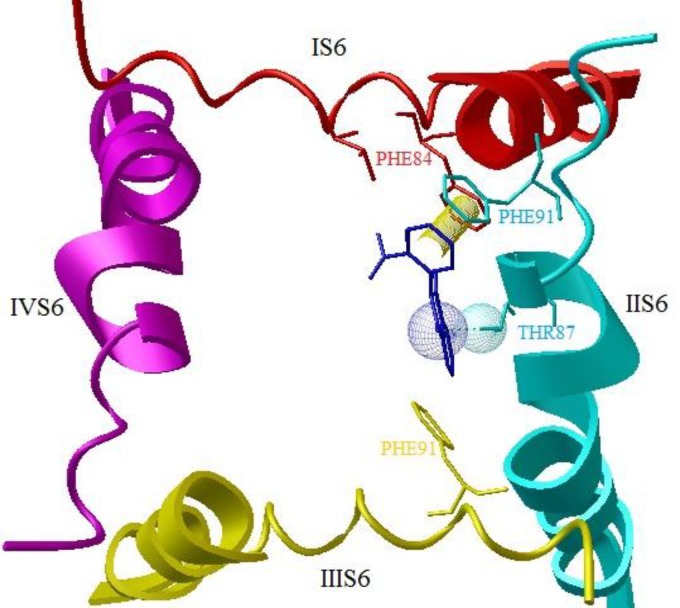
Docked structure of compound 16 in model of the open sodium channel pore, hydrogen bind ‎‎ (distance: 2.082, binding energy: -5.64) is represented by dashed green line between the oxygen ‎of carbonyl of imide and the OH of TYR87, Pi-Pi (π-π) interaction represented as a yellow cylinder ‎that there is an efficient hydrophobic interaction between N-Aryl part of 16 and PHE84 of domain ‎I and a weak interaction between16 and PHE91 of domain II and domain III

Our docking studies reveals while phenytoin interacted with the domain IV-S6 of NaV1.2 (Figure 1), but N-Phenyl Phthalimide derivatives, interacted mainly with the domain II-S6 by making a hydrogen bond and have additional hydrophobic interaction mainly with domains I , II, IV and sometimes with domain III in the channel's inner pore (Figure 2A). This molecular modeling shows that the oxygen in NO_2_ forms a hydrogen bonding interaction with the OH of TYR87 in compounds 8 and 4 (Figure 2B) and with LYS7 in compound 14. The oxygen in carbonyl of imide forms a hydrogen bond with the OH of TYR87 in compounds 2, 7, 15, 16, 17 and 19 (Figure 3). Figure 4 shows a docking prediction in which a hydrogen bond could be formed between the oxygen of COOH group in compound 13 and hydrogen of OH group of THR87 in the active site. In the compounds 9 and 16, there is an efficient Pi-Pi (π-π) interaction between N-phenyl ring and the aromatic ring of PHE84 of domain I (Figure 5). Among these inhibitors, compound 4 which has a nitro moiety in the in position 3 of aromatic ring, shows the most binding energy with a hydrogen binding with domain II of the receptor (distance: 2.089) (Figure 2B). Based on the predicated binding energies (Table 2), some of our designed compounds should be more potent than phenytoin; however, the experimental data did not confirm this, possibly due to low logP of these compounds.

**Table 1:** The structure of docked compounds (1-20)

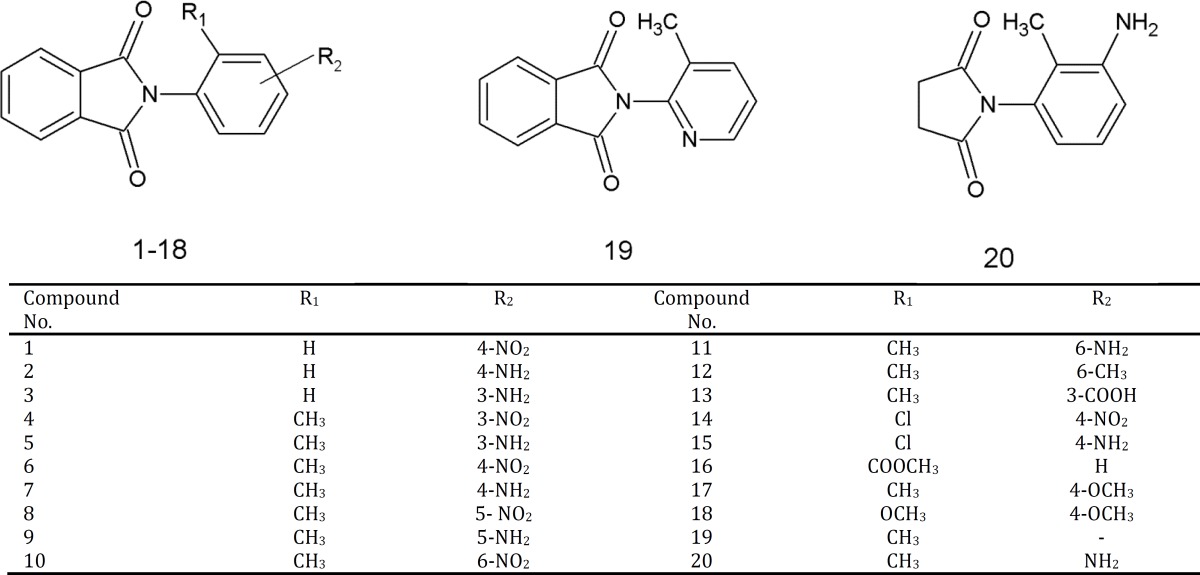

**Table 2:** Docking results of N-Phenyl Phthalimide derivatives using of Auto dock software (version 4)

Com.no.	Binding energy^a^	K_i_ (µm)^ b^	Intermol energy^c^	Electrostatic energy	Total internal energy	Torsional energy	Log P	Docking energy^ d^
1	-5.73	63.02	-6.33	-0.19	-0.35	0.60	2.63	-6.68
2	-5.77	59.12	-6.36	-0.14	-0.31	0.60	1.89	-6.67
3	-5.82	54.44	-6.41	-0.01	-0.32	0.60	1.89	-6.73
4	-6.46	18.46	-7.05	-0.32	-0.48	0.60	3.09	-7.53
5	-5.80	56.25	-6.39	-0.01	-0.39	0.60	2.36	-6.78
6	-6.02	38.89	-6.61	-0.99	-0.27	0.60	3.09	-6.73
7	-5.90	47.64	-6.50	-0.14	-0.45	0.60	2.36	-6.95
8	-6.30	24.13	-6.90	-0.27	0.48	0.60	3.09	-7.38
9	-5.78	58.42	-6.37	-0.04	0.32	0.60	2.36	-6.69
10	-5.54	87.63	-6.13	-0.08	-0.53	0.60	3.09	-6.66
11	-5.40	98.56	-6.06	-0.05	0.48	0.60	2.36	-6.54
12	-5.64	73.91	-5.94	0.00	-0.49	0.30	3.61	-6.43
13	-5.88	48.70	-6.78	-0.31	-0.38	0.89	2.84	-7.16
14	-5.84	52.15	-6.44	-0.95	-0.48	0.60	3.14	-6.92
15	-6.09	34.64	-6.68	-0.14	-0.53	0.60	2.41	-7.21
16	-5.64	73.75	-6.53	0.00	-0.57	0.89	2.40	-7.10
17	-5.65	72.45	-6.25	0.00	-0.52	0.60	2.89	-6.77
18	-5.40	109.4	-6.30	0.00	-0.69	0.89	2.17	-6.99
19	-5.66	71.36	-5.96	-0.06	-0.25	0.30	2.99	-6.21
20	-4.18	868.6	-4.77	-0.04	-0.38	0.60	0.54	-5.15
PHE	-5.83	53.37	-6.43	-0.04	-0.71	0.60	2.08	-6.74

^a ^The predicted binding energy (Kcal/mol) is the sum of intermolecular energy and torsional free energy

^b ^Inhibition constant (K_i_): exp (∆G×1000) / (R_cal_×TK) where ∆G is the docking energy, R_cal_is 1.98719 and TK is 298.15

^c ^Intermolecular energy is sum of Vdw-hb-desolv-energy and electrostatic-energy

^d ^Docking energy is the sum of intermolecular energy and ligand^’^s internal energy

## Discussion

In agreement with binding energies, presence of electronegative and electropositive groups in orto (2) and para (4) position of N-aryl part did not display significant difference in binding energy. However, presence of electronegative moieties in meta (3) position enhances binding energy. Comparison of the compounds 1-19 (phthalimide containing inhibitors) with the compound 20 (succinimide containing inhibitor), reveals that the deletion the phenyl ring from phthalimide reduced binding energy, significantly. Comparison of the compounds 17 with 18 reveals that presence of Cl, CH_3_ and H in position 2 of N-phenyl ring, increase binding energy more than OCH_3 _group.

Based on the pharmacological study, the most potent compound of these series is compound15. In vitro and *in vivo* results have disclosed that compound 15 is more efficacious than phenytoin, ED50 (effective dose in 50% of animals tested) of 5.7 mg/kg, apparent IC_50_ (the 50% maximal inhibitory concentration) of 0.15 µm and PI (protective index) of 48 for compound 15 versus 29.8 mg/kg, 0.93 µm, 6.9 (PI) for phenytoin, respectively (8). In conformity with pharmacological data and docking analysis, comparison of the phthalimide containing inhibitors (e.g. compound 5) with the succinimide containing inhibitor (e.g. compound 20), reveals that the phthalimide affects the pharmacological activity more efficiently than succinamide.

## Conclusion

A Series of N-aryl phthalimide analogues were subjected to docking studies. Our docking studies revealed that most of compounds mainly interacted with the domain II-S6 of NaV1.2 by making a hydrogen bond and have an additional hydrophobic interaction with domain I, II, III and IV in the inner pore of the channel. In compounds 2, 7, 15, 16, 17 and 19 the oxygen of carbonyl group plays a major role in making hydrogen binding interactions with the OH of THR87. In the compounds 8, 4 and 14 having nitro moiety, the oxygen of nitro group has an efficient hydrogen binding interaction with the OH of TYR87 or NH_2_ of LYS7, respectively. The N-aryl part of phthalimide pharmacophore makes a hydrophobic interaction with the hydrophobic pocket of receptor, involving residues PHE84 and PHE91 of domains I and II, respectively. The Aryl part of phthalimide has a weak interaction with the PHE91 of domain III. In compounds 9 and 16, there is an efficient pi-pi interaction between N-aryl part and the aromatic ring of PHE84 of domain I.

 This docking analysis reveals that the phenyl ring of phthalimide which has the main role in drug-receptor interaction should be kept and in order to achieve better potency, an electronegative group can be provided at the meta position of N-aryl part. Currently, the results of this study are being used for the design of newer compound with better anticonvulsant activity.
